# Experience-Specific Dimensions of Consciousness (Observable in Flexible and Spontaneous Action Planning Among Animals)

**DOI:** 10.3389/fnsys.2021.741579

**Published:** 2021-09-10

**Authors:** Angelica Kaufmann

**Affiliations:** Cognition in Action Unit, PhiLab, University of Milan, Milan, Italy

**Keywords:** animal consciousness, action plan, temporal cognition, ethology, comparative psychology

## Abstract

The multidimensional framework to the study of consciousness, which comes as an alternative to a single sliding scale model, offers a set of experimental paradigms for investigating dimensions of animal consciousness, acknowledging the compelling urge for a novel approach. One of these dimensions investigates whether non-human animals can flexibly and spontaneously plan for a future event, and for future desires, without relying on reinforcement learning. This is a critical question since different intentional structures for action in non-human animals are described as served by different neural mechanisms underpinning the capacity to represent temporal properties. And a lack of appreciation of this variety of intentional structures and neural correlates has led many experts to doubt that animals have access to temporal reasoning and to not recognize temporality as a mark of consciousness, and as a psychological resource for their life. With respect to this, there is a significant body of ethological evidence for planning abilities in non-human animals, too often overlooked, and that instead should be taken into serious account. This could contribute to assigning consciousness profiles, across and within species, that should be tailored according to an implemented and expansive use of the multidimensional framework. This cannot be fully operational in the absence of an additional tag to its dimensions of variations: the *experience-specificity* of consciousness.

## Introduction

Cognition varies extensively in nature as individuals adapt to the specific challenges they experience in life (Irwin, 2020). Sumatran and Bornean orangutans, for example, have developed impressive vocal communicative skills because they live in isolation in a very dense arboreal environment in which individuals of a population cannot rely on a visually transmissible communicative repertoire, like gestures. On the contrary, chimpanzees in Uganda and bonobos in DR Congo, do not live in isolation and have developed sophisticated gestural repertoires that they use to communicate. Boesch’s (2021) calls this *experience-specific cognition*. Such considerations over cognition ought to be extended to the study of comparative animal consciousness, a field of research that could be accordingly rebranded as *experience-specific consciousness*.

The recent multidimensional framework to the study of consciousness (Birch et al., 2020), which comes as an alternative to a single sliding scale model, offers a set of experimental paradigms for investigating dimensions of animal consciousness, acknowledging the compelling urge for a novel approach. One of these dimensions investigates whether non-human animals can flexibly and spontaneously plan for a future event, and for future desires, without relying on reinforcement learning. This is a critical question since different intentional structures for action in non-human animals are described as served by different neural mechanisms underpinning the capacity to represent temporal properties (Cai et al., 2012; Mayo and Sommer, 2013; Schormans et al., 2017; Feenders and Klump, 2018; Perry and Chittka, 2019; Viera and Margolis, 2019). And a lack of appreciation of this variety of intentional structures and neural correlates has led experts (Hoerl and McCormack, 2019; Redshaw and Suddendorf, 2020) to doubt that animals can have access to temporal reasoning and to not recognize temporality as a mark of consciousness, and as a psychological resource for their life. With respect to this, there is a significant body of ethological evidence for planning abilities in non-human animals, too often overlooked, and that instead should be taken into serious account. This could contribute to assigning consciousness profiles, across and within species, that should be tailored according to an implemented and expansive use of the multidimensional framework. This cannot be fully operational in the absence of an additional tag to its dimensions of variations: the *experience-specificity* of consciousness.

To appreciate the significant change of perspective that is now encouraging researchers to treat the subject of consciousness in novel terms, it shall be noticed that since not very long ago, consciousness was one of those subjects that researchers were advised not to write about up until tenure. Even now, if one writes about consciousness in non-human animals that person should be ready to face the dubious looks from a lot of skeptics (see Andrews, 2016; Allen and Trestman, 2017, 2020, for a review of arguments for and against animal consciousness from a philosophical and empirical perspective). But the wall of skepticisms toward the legitimacy to write about consciousness, and especially about consciousness in non-human animals began to fall with, courtesy of the Cambridge Declaration on Consciousness (Low et al., 2012). This document assesses that the neurological substrates of all mammals, birds, and many other creatures, including octopuses, are complex enough to support conscious experience. As a result, the first achievement of this change of perspective was the fact that the question was no longer as to whether animals other than humans were conscious, but what their consciousness would look like. The second significant and unprecedented achievement since 2012, was that of seeing researchers acknowledging that to place an organism on a single sliding scale model for consciousness at the top of which—that goes without saying—we would find humans, is a methodological mistake, symptomatic of a widespread tendency resulting from a failure to meet the two following explanatory targets: no-underestimation principle and no-overascription principle. The first one is the principle according to which we should not underestimate the richness of all animal experiences since the neurological substrates for conscious experience are present in a variety of forms among non-human animals. And the second one is the principle according to which we should not overascribe supposedly desirable similarities between non-human animal experience and human animal experience since the neurological substrates of human conscious experience are one among various different neurological structures allowing for conscious experience.

A recent proposal presents itself as an excellent candidate to meet both principles. This is the multidimensional framework to the study of consciousness presented by Birch et al.’s (2020) work, which outlines a set of experimental paradigms for investigating dimensions of animal consciousness, as an alternative to a single sliding scale model. They highlight five significant dimensions of variation within and across animal species: perceptual richness (p-richness), evaluative richness (e-richness), integration at a time (unity), integration across time (temporality), and self-consciousness (selfhood). Taking the case of integration across time will allow for the introduction of an additional tag to these dimensions of variations: the *experience-specificity* of consciousness.

## Consciousness Is Integration Across Time

Various researchers (Osvath and Martin-Ordas, 2014; Müller et al., 2017; Martin-Ordas, 2020; Martin-Ordas et al., 2020; van Leeuwen, 2021) have contributed evidence on the relationship between the experience of time and agency in the specific experience of non-human animals that supports the proposal advanced by Birch, Schnell and Clayton that a multidimensional framework is beneficial to the study of consciousness within the same animal species and across different animal species.

To discuss the relationship between temporal experience and agency, the present focus is on integration across time (temporality), and especially on future planning. When we act, we act across time, and human beings along with many other species, are capable of producing and expressing complex intentional structures for action (Dickinson, 2012). Behavioral manifestations of such complex structures suggest that various creatures possess temporal understanding (Hoerl and McCormack, 2001), but that they cannot reason about time (Hoerl and McCormack, 2019). That is, non-human animals seem able to represent temporal properties such as duration, order of events, causal links between events, and to represent time as passing, but they lack the capacity to understand time as a measure of change (see for example, Blaisdell et al., 2006). However, representing time as a measure of change is an essential aspect of action planning, and thereby providing an account of how different animal species represent time according to their specific experiences is a crucial component in any investigation of their capacity for action planning (Kaufmann, 2015, 2016; Safina, 2016; Kaufmann and Cahen, 2019).

van Schaik et al. (2013) argue that the capacity for action planning relies on two cognitive abilities: self-control and mental time travel. Self-control is understood as the capacity to repress one’s own immediate need and postpone a reward (Osvath and Osvath, 2008; MacLean et al., 2014). Mental time travel is defined as the capacity to mentally represent potential future events (Clayton et al., 2003; Tulving, 2005; Rosati et al., 2007; Roberts and Feeney, 2009; Corballis, 2019). These two core skills that a cognitive system needs to plan future actions are, arguably, complementary. Evidence shows that the capacity that many non-human animals have for mental time travel is at play in a variety of planned actions, such as tool-using practices and anticipatory vocalizations, among other cases (Osvath et al., 2012).

We will look at tool-using first. Chimpanzees can appreciate the difference between present and future uses of the same tool, and they can articulate a coherent sequence of time-displaced intentional actions that involve that object. Since the vast majority of empirical evidence for tools manipulation over time concerns stones, these activities are grouped under the label of “stone handling” behaviors (Cenni et al., 2020). The empirical literature on the matter is flourishing (Bobrowicz et al., 2020). We benefit from various reports on goal-oriented anticipatory behavior like termite fishing and nut-cracking (Boesch and Boesch, 1990; Voelter and Call, 2014), moss-sponging and leaf-sponge re-use (Hobaiter et al., 2014). It is still a matter of controversy whether we can infer instances of action planning from these studies. One reason is that these studies were not meant to investigate planning capacities directly.

The first study that directly addressed a question on action planning capacities, and that provided positive results, shows that orangutans and bonobos can save tools for future use (Mulcahy and Call, 2006); a second study discovered the same abilities in orangutans and chimpanzees as well (Osvath and Osvath, 2008); a third study reinforced these findings with new evidence on chimpanzees (Dufour and Sterck, 2008); and a fourth, but indeed the first agreed upon piece of unambiguous evidence of planning capacities in non-human primates is that recorded by Osvath (2009). This study focused on a captive male chimpanzee (*Pan troglodytes*), named Santino, who was observed (for over 10 years) to have very articulated dominance displays: hurling stones at zoo visitors. The animal would intentionally select, store, conceal, and eventually throw stones at others with the intent of showing dominance. His behavior did not go unnoticed, because even after the zookeepers had cleaned up the compound from every stone, Santino would manage to continue hurling other stones. He started to collect stones from the water moat that surrounds the outside compound. Santino stored them for a later purpose. The chimpanzee behavior has been thus analyzed: the first phase includes the selection, collection, and concealment of the stones. The second phase consists in the manufacturing of discs from concrete, when ready at hand stones were not available. The third phase is the use of these objects as weapons to hurl at zoo visitors. In Osvath and Karvonen (2012), they improved the experimental procedure of their observational studies and reported what follows: the manufactures from concealment become the preferred weapon. The chimpanzee positioned these concealments very close to the visitors’ observation area. He started to deploy a two-step deceptive strategy: firstly, the chimpanzee kept his “weapons” occluded from the visitors’ visual space (see Hare et al., 2001, for evidence that chimpanzees appreciate when something in their visual field is unavailable to someone else’s sight), and secondly, he inhibited his dominance display behavior in order not to scare the visitors and keep them close enough to the observation area. Notably, the chimpanzee had a calm attitude during the first and the second phase, while he got very agitated during the third one—as if he could appreciate the fact that showing arousal from the beginning was going to scare the onlookers ahead of time and compromise the plan.

Osvath classifies this behavior as a planned activity because it is a time-structured intentional action that can be further divided into sub-phases or sub-plans. Santino intends to display dominance, and his plan is a threefold activity extended to the future. Osvath maintains that: “In order for a behavior to signal planning for a future state the predominant mental state during the planning must deviate from the one experienced in the situation that is planned for. The above behavior is clearly identifiable as planning for a future state” (Osvath, 2009, p. 191). The *predominant mental state* is the intentional structure that triggers and subsequently guides the plan throughout its phases. As such, intentions deviate from the mental states that guide the ongoing planned activity at the time it is being experienced. In addition to the threefold structure of the stone hurling planned activity, there are two distinct behaviors to be highlighted: firstly, the chimpanzee’s ability to appreciate whether a given object falls within or outside of the visual field and space of action of a potentially competing third part, and how this affects the structure of the plan of action; secondly, the chimpanzee’s awareness of the fact that repressing its own dominant attitude could bring an advantage toward the achievement of the intended outcome. These two behaviors exemplify the capacity for cross-temporally referential connectivity, individuated by Bratman (1987, 2014), that is the feature of intentions that characterizes these mental states as both backward and forward-looking.

A different observational study by van Schaik et al. (2013) examined the extent to which the direction of long calls emitted by male Sumatran orangutans (*Pongo abelii*) and Bornean orangutans (*Pongo pygmaeus wurmbii*) indicated the direction of their future travel. These animals live in a very dense tropical forest and are semi-solitary, thus often out of sight from other members of their population. The goal of male orangutan’s long calls is that of indicating to female members the future travel direction of the male. Vocalizations are performed by individuals when stationary and can anticipate the direction of their travel 1 day ahead. The study of van Schaik et al. (2013) focused on three issues: first, they tested whether the direction in which flanged male Sumatran orangutans give spontaneous long calls generally predicts the subsequent travel direction. Second, they investigated whether a new spontaneous long call indicates the subsequent travel direction better than the old one would have if no new call had been given. Third, they tested the extent to which long calls given in the evening at or near the night nest still indicate travel direction during the next day, thus indicating future planning independent of the current motivational state. The temporal dimension of consciousness is particularly interesting with respect to the evidence at hand about the capacity displayed by male Sumatran orangutans and Bornean orangutans to communicate their future travel directions and the corresponding ability displayed by female orangutans to be receptive to such communicative intentions (van Schaik et al., 2013; Spillman et al., 2015; Askew and Morrogh-Bernard, 2016; Lameira and Call, 2018). As described, together tool-use and travel calls provide fertile ground for discussing integration across time as a marking dimension for a consciousness profile. Yet, this type of evidence is not properly acknowledged within the multidimensional framework.

## Integration Across Time Is Best Observed in Flexible and Spontaneous Behavior

The multidimensional framework and its current experimental paradigms can be informed by implementing the empirical literature, currently deployed, with more evidence from ethology, in addition to evidence from comparative experimental psychology. In particular, as said, this analysis focuses on evidence that emphasizes the presence in non-human primates of the capacity for integration across time and temporal reasoning. To explain why ethology matters in this context, I shall discuss this dimension of consciousness in terms of the Lean Temporal Integration Approach and Rich Temporal Integration Approach. The multidimensional framework buys elements of both approaches, reasonably so. The first and fundamental difference between the two is given by methodology. The Lean Temporal Integration Approach is built on the research methods of comparative experimental psychology, that is, behavioral experiments run in artificial settings (Tomasello and Call, 1997; Leavens et al., 2010; Webster and Rutz, 2020); the Rich Temporal Integration Approach is the result of the research methods of cognitive ethology, that is, research in the field, mostly done as observation of animal behavior in the wild (Nishida et al., 1983; Healy et al., 2009; Smulders et al., 2010; Janmaat et al., 2014, 2016; Rosati, 2017; Boesch, 2020, 2021; Bräuer et al., 2020). These two approaches lead to very different conclusions about the structure of non-human animal experience: the Lean Integration Approach argues for a lack of motivation in pursuing action planning on the side of the animal, and from this lack of motivation it infers a lack of cognitive faculties that are needed to act spontaneously toward a future goal. Conversely, the Rich Integration Approach distinguishes evidence for lack of motivation to interpretations about lack of cognitive capacities. When the comparative experimental psychologist asks the question of what a certain species is capable of achieving in terms of spontaneous future goals, she is investigating the motivational aspect of instances that can reflect this behavior. When the ethologist asks the question of what is possible to achieve in terms of spontaneous future goals, she is investigating the behavioral criteria that can account for this cluster of flexible action plans. I argue that the two claims of the Lean Temporal Integration Approach can and ought to be kept separate: evidence that non-human animals are mostly pursuing repetitive activities motivated by recurrent goals is not evidence that they are only capable of pursuing recurrent goals. Evidence that non-human animals appreciate the recurrent nature of the goals of others is not evidence that they are capable only of ascribing recurrent goals to others. I concede to the Lean Temporal Integration Approach that the vast majority of non-human animals activities is driven by recurrent goals and by the capacity to ascribe recurrent goals to others; what I disagree with, in the context of the Lean Temporal Integration Approach is the assumption that this capacity to form and ascribe recurrent goals is limited to recurrent goals. Evidence from empirical research in support of the Rich Temporal Integration Approach points to the fact that non-human animals are capable of forming and ascribing spontaneous and flexible goals that extend to articulated actions. The purpose of presenting these two approaches is to highlight the fact that the analysis of the five dimensions of variation should be sensitive as to whether the evidence taken into account at a time is obtained from observational work or from a controlled environment. A consciousness profile of a given animal species drawn from evidence from ethology, would in all likelihood differ from one tailored from evidence from comparative experimental psychology.

I have exemplified this methodological difference between the two approaches by focusing on specific observational studies. Out of the various empirical evidence ascribing consciousness to non-human animals, I turned attention to evidence from ethology, which are revealing of the richness of animal cognition, crucial to consciousness and made manifest by the spontaneity and flexibility of action (Pennartz et al., 2019). I wanted to explain how, through a Rich Temporal Integration Approach, consciousness can be observed in various species and how non-human animals can be assigned a consciousness profile tailored according to the specificity of their experience.

## Conclusion

The analysis of the dimensions of variation should be sensitive to whether the evidence taken into account at a time is obtained from observational work or from a controlled environment. As explained, a multidimensional framework and its current experimental paradigms can be informed by implementing the empirical literature with more evidence from ethology, in addition to evidence from comparative experimental psychology.

Conscious experience is assessed through a series of behavioral, cognitive and neurological criteria. Firstly, contrary to what most people assumed until a decade ago, the impossibility of collecting verbal reports from animals does not preclude the scientific investigation of animal consciousness. It is not only animals that are incapable of providing verbal reports about their inner life, but also young children and patients in minimally conscious states. And since most people will not deny conscious experience to children or such patients, so they should not deny conscious experience to other animals. Secondly, to deploy a single sliding scale model for measuring consciousness, would amount to following a fallacious methodology and a hardly scientific one, not least, as just said, because conscious experience cannot and should not be investigated according to rigid criteria such as verbal reports. For these reasons, the behavioral, cognitive and neurological criteria for conscious experience should be sensitive to dimensions of variation that should exist within a multidimensional framework conceived in order to provide a different consciousness profile for each animal species.

In particular, as discussed, consciousness can be observed in the flexible and spontaneous planning behavior of various primates, and these animals can be given a consciousness profile tailored according to an implemented and expansive use of the multidimensional framework which ought to take into account an additional tag to its dimensions of variations: the *experience-specificity* of consciousness.

## Data Availability Statement

The original contributions presented in the study are included in the article/supplementary material, further inquiries can be directed to the corresponding author/s.

## Author Contributions

The author confirms being the sole contributor of this work and has approved it for publication.

## Conflict of Interest

The author declares that the research was conducted in the absence of any commercial or financial relationships that could be construed as a potential conflict of interest.

## Publisher’s Note

All claims expressed in this article are solely those of the authors and do not necessarily represent those of their affiliated organizations, or those of the publisher, the editors and the reviewers. Any product that may be evaluated in this article, or claim that may be made by its manufacturer, is not guaranteed or endorsed by the publisher.
